# The Location and Nature of General Anesthetic Binding Sites on the Active Conformation of Firefly Luciferase; A Time Resolved Photolabeling Study

**DOI:** 10.1371/journal.pone.0029854

**Published:** 2012-01-17

**Authors:** Sivananthaperumal Shanmugasundararaj, Simon Lehle, Herve I. Yamodo, S. Shaukat Husain, Claire Tseng, Khanh Nguyen, George H. Addona, Keith W. Miller

**Affiliations:** 1 Department of Anesthesia and Critical Care, Massachusetts General Hospital, Boston, Massachusetts, United States of America; 2 Department of Biological Chemistry and Molecular Pharmacology, Harvard Medical School, Boston, Massachusetts, United States of America; Emory University, United States of America

## Abstract

Firefly luciferase is one of the few soluble proteins that is acted upon by a wide variety of general anesthetics and alcohols; they inhibit the ATP–driven production of light. We have used time–resolved photolabeling to locate the binding sites of alcohols during the initial light output, some 200 ms after adding ATP. The photolabel 3-azioctanol inhibited the initial light output with an IC50 of 200 µM, close to its general anesthetic potency. Photoincorporation of [^3^H]3-azioctanol into luciferase was saturable but weak. It was enhanced 200 ms after adding ATP but was negligible minutes later. Sequencing of tryptic digests by HPLC–MSMS revealed a similar conformation–dependence for photoincorporation of 3-azioctanol into Glu-313, a residue that lines the bottom of a deep cleft (vestibule) whose outer end binds luciferin. An aromatic diazirine analog of benzyl alcohol with broader side chain reactivity reported two sites. First, it photolabeled two residues in the vestibule, Ser-286 and Ile-288, both of which are implicated with Glu-313 in the conformation change accompanying activation. Second, it photolabeled two residues that contact luciferin, Ser-316 and Ser-349. Thus, time resolved photolabeling supports two mechanisms of action. First, an allosteric one, in which anesthetics bind in the vestibule displacing water molecules that are thought to be involved in light output. Second, a competitive one, in which anesthetics bind isosterically with luciferin. This work provides structural evidence that supports the competitive and allosteric actions previously characterized by kinetic studies.

## Introduction

**Figure 1:**
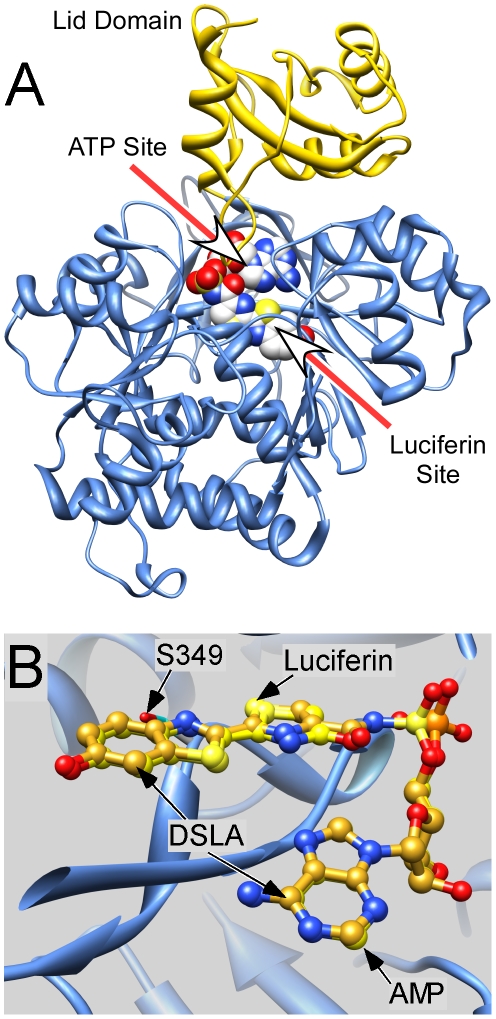
The structure of Japanese luciferase. **A.** The backbone structure of the highest resolution structure (2D1S.pdb) is shown in ribbon format. The lid domain is shown in gold. The two ligand–binding sites are indicated with arrows and are shown occupied by the concatenated active state analog DSLA. **B.** The benzothiazole ring of oxyluciferin and DSLA are isosteric. The structures of Japanese luciferase with oxyluciferin +AMP bound (2D1R.pdb) and with the concatenated active state analog DSLA bound (2D1S.pdb) were superimposed. The backbone structures overlay exactly in this region and only that for 2D1R is shown. The ligands' atoms are color coded normally except carbons, which are goldenrod for DSLA and yellow for oxyluciferin and AMP.

It remains a challenge to understand how the function of a protein can be affected when a low affinity drug such as a general anesthetic binds to it. While such an understanding may come from structures of general anesthetics bound to proteins, general anesthetics often act selectively on transient states of receptors such as open channels [1]. While some progress is being made with ion channels [2], it is often unclear what conformation of a channel has been crystallized and what relationship it bears to that channel in a native environment. Thus, currently detailed mechanisms of anesthetic action are more likely to emerge from studies of soluble proteins. The choice of model is constrained because of the structural diversity of general anesthetics [3], and the observation that a given protein often only interacts with a narrow range of anesthetics [4]. A suitable model is luciferase because it is inhibited by a fairly wide range of general anesthetics and has long been a focus of mechanistic studies. It is readily available and its activity is easily monitored by its light output [5], [6], [7], [8], [9]. The structure of firefly luciferase (Fig. 1A) has been determined in a number of conformations [10], [11], [12]. Luciferase contains two binding pockets in close structural proximity; one for ATP and the other for the substrate luciferin in firefly luciferase (Fig. 1B. The enzyme catalyzes the emission of light from its substrate by a series of reactions using Mg-ATP and oxygen. The bioluminescence reaction proceeds via the initial formation of an enzyme-bound luciferyl-adenylate intermediate (modeled by 5′-O-[*N*-(dehydroluciferyl)- sulfamoyl]adenosine (DSLA; Fig. 1B) that is oxidized with the formation of an excited α-peroxylactone intermediate, AMP and carbon dioxide. A photon is emitted during relaxation of the lactone from the excited state to form oxyluciferin [11].

It has been hypothesized that anesthetics act on luciferase by competing for the binding site of the substrate involved in light emission. They compete with an aldehyde in bacterial luciferase [8] and luciferin in the firefly luciferase [9]. When firefly luciferase and luciferin are rapidly mixed with ATP, the initial output of light is inhibited by general anesthetics. The dependence of this inhibition on luciferin concentration appears to be competitive. However, the limited solubility of anesthetics always makes it impossible to perform such studies over a sufficiently wide concentration range to completely rule out the alternative mechanism of a negative heterotrophic or allosteric action [13]. Indeed, greater complexity is hinted at in a number of studies. In a steady state kinetics study on firefly luciferase, alkanols were found to act allosterically whereas their fatty acid analogs acted competitively [14], [15]. More recently, the determination of a crystal structure containing bromoform bound to the unliganded American firefly luciferase, a conformation which has low affinity for anesthetics [16], led to a more detailed kinetic analysis [17]. At high luciferin concentrations, which surmount the competitive inhibition, it was apparent that bromoform was still able to inhibit light emission presumably by an allosteric action.

The aim of the present work was to locate the anesthetic sites of action on firefly luciferase during the initial few hundred milliseconds of ATP–induced light emission. To do so we have employed diazirine derivatives of alkanols and benzyl alcohol and time–resolved photolabeling to show that the degree of photoincorporation varies with the conformational state of luciferase. The photolabeled residues are mainly clustered in or near the luciferin pocket within a region of luciferase whose structure has recently been shown to vary during the catalytic cycle [11], [12].

## Materials and Methods

### Anesthetic Potency

General anesthetic potency was assessed with institutional approval (Protocol #: 2006N000124/4. Research Animal Care, Massachusetts General Hospital, 149 13th Street, Charlestown, MA 02129,Tel: (617) 726-3495) in pre-limb bud Xenopus tadpoles, 1.5–2 cm in length (Xenopus 1, Inc., Dexter, MI) as previously described [18].

### Materials

Recombinant luciferase derived from *Luciola cruciata* (Japanese firefly) was purchased from Wako Chemicals USA Inc. (Richmond, VA). Recombinant luciferase derived from *Photinus pyralis* (North American firefly) was purchased from Sigma-Aldrich (St. Louis, MO). Luciferin and adenosine 5′-triphosphate (ATP) were obtained from Sigma-Aldrich. 3-Azibutanol, 3-azioctanol and 7-azioctanol were synthesized as previously described [19], [20]. TFD-benzyl alcohol ((4-(3-(trifluoromethyl)-3*H*-diazirin-3-yl)phenyl)methanol) was synthesized using Husain's method [18]. [^3^H]-3-azioctanol was prepared from 3-azioctanol by American Radiochemical Company (St Louis, MO). Glycylglycine was obtained from EMB Bioscience Inc. (La Jolla, Ca). Magnesium sulfate (MgSO_4_) was obtained from J.T. Baker Inc. (Phillipsburg, NJ). Sequencing-grade trypsin was purchased from Promega, Madison, WI. All other reagents were obtained from Sigma-Aldrich (St. Louis, MO).

### Photolabeling of luciferase

Luciferase was photolabeled in the presence or absence of ATP in two time domains: after a 30 min pre-incubation with all ligands, and 200 ms after rapidly mixing the enzyme with photolabels (time-resolved photolabeling). For equilibrium labeling, recombinant luciferase (4.3 µM) was equilibrated for 30 min with 10 or 100 µM of ligands in buffer-A (50 mM N-glycylglycine and 10 mM MgSO_4_, pH 7.8), with or without 2 mM ATP. For each condition (1 or 2), the prepared reaction mixture was distributed in three wells of a 96-well glass plate (Zinsser Analytic, Northridge, CA) kept on ice to protect from thermal side reactions and irradiated for 30 min at 365 nm with a Black-Ray UV lamp (model UVL-56 Upland, CA) at a distance of ∼1 cm. Photolabeling was quenched with an equal volume of 2X SDS-PAGE sample loading buffer. The photolabeled protein was separated from the reaction mixture by SDS-PAGE.

For time-resolved photolabeling of luciferase at 200 ms after addition of ATP, we adapted a method from the time-resolved photolabeling of *Torpedo californica* nicotinic receptor as previously described [21]. Briefly, the loop of one of the two six-way sample valves was filled with 0.5 mL of purified recombinant luciferase (4.3 µM) equilibrated with the photolabels in buffer-A. The other six-way sample valve's loop was filled with 0.5 mL of the photolabels in buffer-A with or without 2 mM of ATP. The contents of the loops were forced through the rapid mixer (<1 ms) by a pneumatic pump and then incubated in an appropriate aging loop for ∼200 ms and expelled onto a rotating stainless steel disk (60 rpm) pre-cooled in liquid nitrogen, where they were instantaneously (<1 ms) frozen in a thin film. The freeze-quenched samples were then irradiated for 30 min at 365 nm as above on the slowly rotating disk (3 rpm) in contact with liquid nitrogen before being melted into sample buffer as above. Three replicates were acquired for each set of conditions, and all experiments were repeated on a separate day.

To quantify photolabel incorporation, luciferase was incubated with [^3^H]3-azioctanol alcohol and the amount of radioactivity incorporated was counted. For experiments with radiolabeled alcohols, the luciferase band from each sample was visualized by Coomassie blue stain, the band was excised, transferred to tubes containing ∼4 mL of gel solubilizer, allowed to stand until the count was stable (typically for 6 days) at room temperature, and assayed by scintillation counting (1900 CA, Tri-Carb, Liquid Scintillation Analyzer, Canberra Packard Instruments Co., Downers Grove, IL). The background radioactivity due to unreacted [^3^H]3-azioctanol label present in the gel was measured from the sections lacking protein below the protein band.

### In-gel proteolytic digestion

In-gel proteolytic digestion was carried out following the method of Shevchenko and Shevchenko [22] to ensure that protein was free from small molecule impurities prior to submission for mass spectrometry analysis. Approximately 10 µg of photolabeled firefly luciferase was separated from other materials by SDS-polyacrylamide gel electrophoresis in a 10% polyacrylamide gel. The Coomassie blue stained luciferase band was cut into ∼2 mm squares, incubated overnight at room temperature with 200 µL of 50 mM ammonium bicarbonate solution, and then washed with acetonitrile for 10 min. The solvent was removed under vacuum, and the gel pieces were rehydrated with aqueous 50 mM ammonium bicarbonate solution containing 12.5 ng/mL modified sequencing-grade trypsin at 4°C. After 45 min of digestion, the excess proteolysis solution was removed and replaced with a volume of 50 mM ammonium bicarbonate solution sufficient to cover the gel for 16–20 hrs overnight incubation at 37°C. Peptides were then extracted from the gel pieces by first removing the ammonium bicarbonate solution and then performing two consecutive 20 min incubations in a solution composed of 50% v/v acetonitrile and 5% v/v formic acid. The extracts were combined, dried under vacuum for ∼1 h and stored at 4°C until analysis.

### Stopped-flow kinetic studies

Stopped-flow kinetic experiments were performed with a BioS X-17 MV spectrofluorimeter (Applied Photophysics, Leatherhead, England). Light output was monitored without filtration. All experiments were carried out in buffer-A at 22±1°C. ATP (2 mM; final concentration) was rapidly mixed with luciferase (20 nM) that had been pre-incubated with luciferin (28 µM) and appropriate concentrations of 3-azioctanol for 30 min. The burst of light for each condition was recorded; the slope of the linear region of the curve was used to characterize the level of the luciferase activity.

### Tandem HPLC and mass spectrometry

LC-MSMS experiments were performed on an Orbitrap mass spectrometer (ThermoElectron, Waltham, MA). This is a fully integrated hybrid mass spectrometer consisting of a linear ion trap mass spectrometer combined with a Fourier transform ion cyclotron resonance mass spectrometer. A nanoscale reverse-phase HPLC capillary column was created by packing 5 µm C18 spherical silica (pore size 200 Å) into a 12 cm fused silica capillary tube of 125 µm inner diameter with a flame-drawn tip fabricated in-house. Peptides stored after trypsin digestion were reconstituted in 5 µL of an aqueous solution composed of 2.5% v/v acetonitrile, 0.1% v/v formic acid and were eluted by a linear concentration gradient (acetonitrile with 0.1% formic acid increasing from 10 to 38% over 30 min followed by a step increase to 100%).

Each eluted peptide was subjected to electrospray ionization. The m/z of each isolated peptide was determined to high accuracy by Fourier transform mass spectrometry (FTMS). The remainder of the sample was fragmented by collision with an inert gas and its tandem mass spectrum (MSMS) was analyzed to determine its sequence using the top down method. After a full MS scan, the top ten most intense peaks were selected for MSMS analysis with a range of 1.5 m/z and with dynamic exclusion on. The position of photoincorporation was determined using the Bioworks browser 3.1 module of the program Xcalibur (ThermoElectron, Waltham, MA).

Peptide fragments will only be detected if they are charged. For cleavage at the peptide bond, when this charge is retained on the N-terminal or C-terminal fragment, the ion is called a b-ion or a y-ion respectively (http://www.ionsource.com/tutorial/DeNovo/DeNovoTOC.htm).

### Molecular modeling

Figures shown in this manuscript were modeled in the program Chimera using structures downloaded from the remediated pdb (http://www.rcsb.org/pdb/home/home.do). To model ethylene glycol in the luciferase vestibule (Fig. S4), we downloaded 3IEP.pdb and its electron density map from the Protein Data Bank into the program Coot [23]. We generated the coordinates of ethylene glycol using the SMILES string C(CO)O, and visually placed it in the excess density using the translate and rotate functions in Coot.

## Results

### Inhibition of luciferase activity by 3-azioctanol

**Figure 2:**
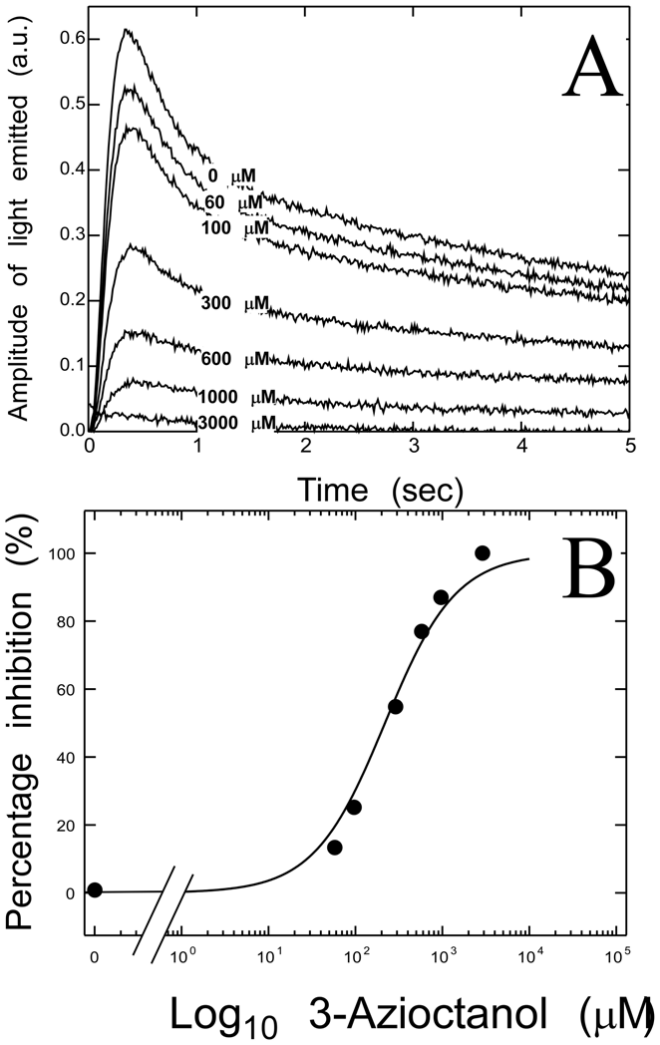
The 3-Azioctanol inhibits ATP-induced luciferase activity. Fresh American firefly luciferase pre-incubated with luciferin and increasing concentrations of the alkanol were rapidly mixed with ATP (see Results). **A.** Increasing concentrations of 3-azioctanol inhibited the initial phase of light emission. **B.** The slope of the linear portion of the initial peak of each curve was normalized (see Results) and plotted against the 3-azioctanol concentrations. Nonlinear least squares fitting yielded an IC_50_ value of 220±47 µM and a Hill coefficient of 1.1±0.2.

In order to assess whether 3-azioctanol, like octanol and other alkanols, inhibits luciferase, American firefly luciferase (20 nM) was pre-incubated for 15 minutes with 0 to 6000 µM 3-azioctanol in the presence of 28 µM luciferin at 22±1°C. These samples were then rapidly mixed with 4 mM ATP in the stopped flow spectrofluorimeter. Final concentrations were half those given above. The light emitted was monitored for 5 s. It showed an initial lag of ∼50 ms, rose to a peak at ∼300 ms and fell slowly for 5 s (Fig. 2A). Increasing concentrations of 3-azioctanol reduced the maximum light output without altering the overall pattern of time–dependence. We used the slope of the linear region of the kinetic curve (∼65 to ∼200 ms) as a measure of inhibition. The data were normalized between the control and 3000 µM slopes and fitted to a logarithmic equation by nonlinear least squares (Fig. 2B) to yield an IC_50_ of 200±46 µM and a slope of 1.0±0.2. For comparison, the general anesthetic EC50 for 3-azioctanol is 160 µM [20]. Under similar conditions, 225 µM 1-octanol inhibited luciferase by ∼50%. This compares to a reported IC_50_ of 280 µM for 1-octanol [16]. 3-Azibutanol (1 mM) also inhibited luciferase. Thus, azialkanols behave like other 1-alkanols with respect to inhibition of luciferase.

### Equilibrium photolabeling of luciferase by [^3^H]3-azioctanol

**Figure 3:**
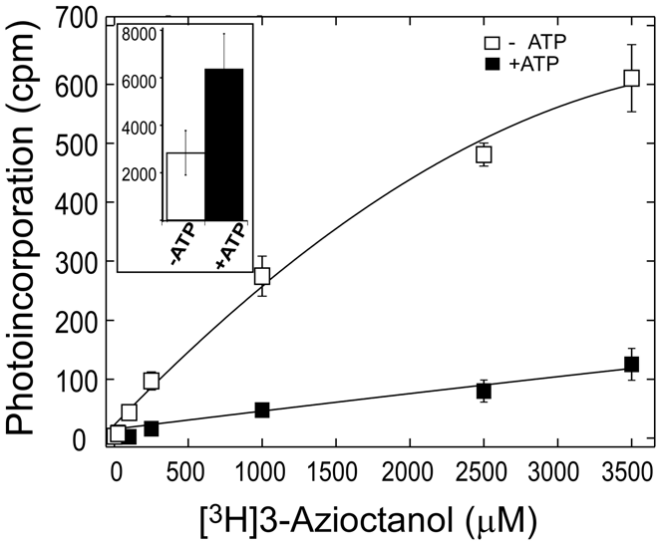
ATP modulates the photoincorporation of [^3^H]-3-Azioctanol into luciferase at equilibrium. Increasing concentrations of [^3^H]3-azioctanol were preincubated for 30 min with 4.3 µM Japanese firefly luciferase in buffer-A containing 0 (□) or 2 mM (▪) ATP, photolabeled and analyzed as described in the text. Each sample was prepared in triplicate and each point represents the mean and standard deviation. Where error bars are not shown, they are smaller than the symbol. **Inset**: Luciferase (4.3 µM) pre-incubated with 1 µM [^3^H]3-azioctanol was rapidly mixed with buffer-A containing 0 (white bar) or 2 mM (black bar) ATP, freeze-quenched after 200 ms and photolabeled (see text). Three shots were acquired for each set of condition and the bars represent the mean and the error bars the standard deviation.

Because the inhibition of luciferase activity by alcohol is ATP-dependent [16], we determined the effect of ATP on the photoincorporation of [^3^H]3-azioctanol. Increasing concentrations of [^3^H]3-azioctanol were preincubated for 30 min with 4.3 µM Japanese firefly luciferase in the absence or presence of 2 mM ATP, and photolabeled as described above. In the presence of ATP, the level of photoincorporation increased linearly (Fig. 3), consistent with either a nonspecific action or binding to sites with very low affinity. In the absence of ATP, photoincorporation was higher at all alcohol concentrations and the shape of the curve suggested saturable binding with a dissociation constant above 1 mM. Similar data were obtained with American firefly luciferase. Thus, at equilibrium, ATP inhibits photoincorporation of [^3^H]3-azioctanol into firefly luciferase.

### Time resolved photolabeling with [^3^H]3-Azioctanol

At first sight the ATP-induced decrease in photoincorporation of [^3^H]3-azioctanol observed above contradicts the allosteric hypothesis of Moss *et al* [16], which predicts that ATP induces a higher affinity binding of alcohols. However, their hypothesis refers to the initial phase of the reaction (≤300 ms), not at equilibrium. Accordingly, we determined photoincorporation into luciferase 200 ms after the addition of 2 mM ATP using time resolved photolabeling. Because of the large sample size required by the apparatus and the cost of tritiated ligands, only a single concentration of [^3^H]3-azioctanol (1 µM) was examined. The data presented in Fig. 3 (inset) show that, unlike at equilibrium, ATP doubles the photoincorporation of [^3^H]3-azioctanol into luciferase at 200 ms. Analysis of the data by the Student's t-test yielded a p-value of 0.002 indicating that the difference in photoincorporation observed without ATP (white bar) and with ATP (black bar) is statistically significant.

### Identification of residues photolabeled in luciferase

To identify residues in the anesthetic binding site(s), we photolabeled Japanese luciferase in both the presence and absence of ATP (at 200 ms and at equilibrium) with the diazirinyl n-alkanols (3-azibutanol, 3- and 7-azioctanol) and with TFD-benzyl alcohol. All samples were proteolytically digested and subjected to HPLC-MSMS. We were able to sequence 94% of the Japanese luciferase, but our data (see below and Tables 1 & 2) show that photolabeling is confined to a few major peptides together with a number of nonspecific sites. Furthermore, to enhance our ability to map the binding sites we used both aromatic and aliphatic alcohols containing diazirine groups because these two classes of agent have different photoselectivities. Although no formal survey has been published, aromatic and aliphatic diazirines react selectively with nearly complementary sets of residues (roughly Leu, Ile, Ser, Met Val, Phe vs. Tyr, Glu, Asp, His, Met, respectively) [24].

**Table 1:** Residues photolabeled in a tryptic fragment of Japanese Firefly Luciferase (306-YDLSNLV**EI**A**S**GGAPLSK-323)^a^ identified by LTQ–FT mass spectrometry and MSMS.

Agent	Alcohol µM	ATP	Time ms	Residue Photolabeled	Charge	*R*_t_, min	X_corr_	bMH^+^	Accuracy^c^ppm
Parent peptide^d^					+2	24.0	6.0	1835.05	0.91
7-azioctanol	100	+	200	E313^f^	+2	31.2	5.5	1963.17	0.79
	100	−	∞	E313	+2	26.1	5.4	1963.17	0.79
	100	+	∞		Photolabeled peptide rarely detected				
3-azioctanol	100	+	200	E313	+2	29.1	5.9	1963.17	0.79
	100	−	∞	E313	+2	28.1	5.4	1963.17	0.79
	100	+	∞		Photolabeled peptide rarely detected				
3-azibutanol	1000	+	200	E313^f^	+2	26.6	5.6	1907.05	0.91
	1000	−	∞	E313	+2	23.5	6.1	1907.05	0.91
	1000	+	∞		Photolabeled peptide not detected				
TFD-benzyl alcohol	100	+	200	I314	+2	30.5	4.4	2022.00	1.96
	100	+	200	S316	+2	29.3	5.4	2022.00	1.96
	100	−	200	I314	+2	31.2	5.6	2022.00	1.96

a Photolabeled residues are indicated in bold.

b Representative observed masses that include increases due to photolabeling by 3-azioctanol and 7-azioctanol (128 Da) or 3-azibutanol (72 Da) or TFD-benzyl alcohol (188 Da).

c Accuracy is the difference between the calculated and observed mass expressed in ppm.

d Parent peptides are those derived from that fraction of Luciferase in a given run that was not photolabeled during an experiment with azialcohols or TFD-benzyl alcohol. See “Materials and methods” section for other details.

**Table 2:** Residues photolabeled in a tryptic fragment of Japanese Firefly Luciferase (340-QGYGLTETT**S**AIIITPEG**D**DKPGASGK-366)^a^ *^n^* identified by LTQ–FT mass spectrometry and MSMS.

Agent	Alcohol µM	ATP	Time ms	Residue Photolabeled	Charge	*R*_t_, min	X_corr_	bMH^+^	Accuracy^c^ppm
Parent peptide^d^					+3	25.8	5.5	2707.93	0.58
7-azioctanol	100	+	200		Photolabeled peptide not detected				
	100	−	∞	D358	+3	21.8	6.0	2836.05	0.70
	100	+	∞		Photolabeled peptide rarely detected				
3-azioctanol	100	+	200		Photolabeled peptide not detected				
	100	−	∞	D358	+3	22.7	5.5	2836.05	0.70
	100	+	∞		Photolabeled peptide not detected				
3-azibutanol	1000	+	200	D358	+3	20.3	5.8	2779.93	0.58
	1000	−	∞	D358	+3	17.3	6.2	2779.93	0.58
	1000	+	∞		Photolabeled peptide not detected				
TFD-benzyl alcohol	100	+	200	S349^e^	+3	24.8	5.0	2894.39	0.96

a–d Same as Table 1.

e Photoincorporation was either in Ser-249 or Ala-250. The latter residue is rarely labeled by aromatic diazirines.

We also photolabeled the American firefly luciferase at equilibrium with the azialkanols, and found the labeling pattern to be identical. The American and Japanese firefly luciferases have both been used in anesthetic studies; the American has been studied extensively, but the highest resolution structure is for the Japanese. Overall, these two proteins are highly homologous (67%), and we have used the Japanese numbering system in this discussion (for the residues of interest, subtracting 2 from the Japanese residue number usually gives that of the American). The two luciferases sometimes differ in their proteolytic cleavage patterns, and we have found this useful (see below).

### Residues photolabeled on peptide Tyr-306– Lys-324

**Figure 4:**
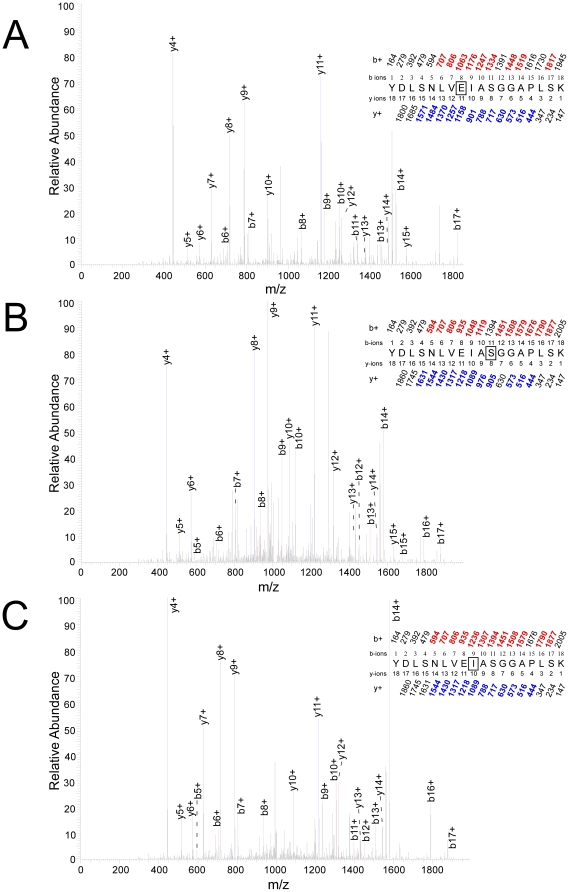
Identification of photolabeled residues in the peptide Tyr-306– Lys-324. The site of photoincorporation of photolabels was inferred from these MSMS spectrum. In the insets, the predicted charge/mass ratios of ions with an intact N-terminus (b-ions) or C-terminus (y-ions) are shown above and below the sequence, respectively, with the indicated charge. The photolabeled residue is boxed and the experimentally observed values are colored (b-ions in red and y-ions in blue) and in bold and their position indicated on the spectrum. Luciferase was photolabeled after exposure to ATP (2 mM)+photolabel for 200 ms. **A.** 3-azioctanol (100 µM) photolabels Glu-313. **B.** TFD-benzyl alcohol (100 µM) photolabels Ser-316. **C.** TFD-benzyl alcohol (100 µM) photolabels Ile-314.

The Japanese luciferase was exposed to ATP and 100 µM 3-azioctanol for 200 ms, rapidly frozen and photolabeled. The samples were treated as described in Methods, and the tryptic fragments analyzed by HPLC-MSMS. A doubly charged peptide of mass 1963.1679 Da with a retention time of 29.1 min was detected by FTMS. This peptide was identified as the 18–residue peptide YDLSNLVEIASGGAPLSK beginning at Y306 and incorporating a single molecule of azioctanol. It was fragmented by collision with an inert gas to yield peptide fragments with an intact N-terminus, called b-ions, or an intact C-terminus, called y-ions. Fig. 4A shows the MSMS spectrum that was used to identify the point of photoincorporation as Glu-313. A sequence of mostly strong singly charged y-ions from y15 to y4 was observed with a loss of 3-azioctanol between y11 and y10. This conclusion was consistent with the b-ion pattern where a run of peaks was observed from b14 to b6 (except b12), with loss of 3-azioctanol between b8 and b7. This assignment had an X_corr_ value of 5.9 (Table 1). Similar results were obtained for Japanese luciferase labeled with 100 µM 7-azioctanol and 1 mM 3-azibutanol at equilibrium in the absence of ATP (Table 1; Figs. S1 & S2). The percent of observed peptides that were labeled was 25–50% at 200 ms after adding ATP, and simultaneous addition of luciferin reduced this to 0–10%. At equilibrium, photolabeling of Glu-313 could be resolved at concentrations as low as 10 µM 3-azioctanol (Fig. S3) and 100 µM 3-azibutanol.

When Japanese luciferase was photolabeled with 100 µM TFD-benzyl alcohol after incubation for 200 ms with ATP, a major photolabeled trypsin product that eluted from the HPLC at 29.3 min was a doubly charged peptide of mass 2021.9993 Da. It was identified as an 18–residue peptide YDLSNLVEIASGGAPLSK, starting at Y306, photolabeled by a single TFD-benzyl alcohol. Fig. 4B shows the MSMS spectrum that was used to identify the point of photoincorporation as Ser-316. A sequence of mostly strong singly charged y-ions from y15 to y4 with the exception of y7 was observed with a loss of TFD-benzyl alcohol between y8 and y6. This result is consistent with the b-ion pattern where a run of peaks was observed from b17 to b5 with the exception of b11, with loss of TFD-benzyl alcohol between b12 and b10. Thus, the photolabeled residue is Ser-316. This assignment had an X_corr_ value of 5.39 (Table 1).

From the same experiment, another tryptic peptide, eluting a little later (30.5 min), was doubly charged with the same mass of 2021.9993 Da, but it was not photolabeled at Ser-316. The MSMS spectrum shows a run of mostly strong singly charged y-ions from y14 to y4 with a loss of TFD-benzyl alcohol between y10 and y9, placing the site of photoincorporation at Ile-314 (Fig. 4C). This conclusion was consistent with the b-ion pattern where a run of peaks was observed from b6 to b14, with loss of TFD-benzyl alcohol between b9 and b8. This assignment had an X_corr_ value of 4.43 (Table 1). Consistent with our observation that TFD-benzyl alcohol photolabeled both Ile-314 and Ser-316, we found that double labeling predominated over single labeling by fourfold with +ATP at 200 ms, but the quality of these double labeled spectra were inadequate to resolve their sites of photoincorporation. The frequency of photoincorporation was lower than with the azialkanols, consistent with the known low photolabeling efficiency of aromatic diazirines, making it difficult to resolve small change in photoincorporation, but simultaneous addition of both ATP and luciferin completely abolished photoincorporation into both residues.

### Residues photolabeled on peptide Gln-340 – Lys-366

**Figure 5:**
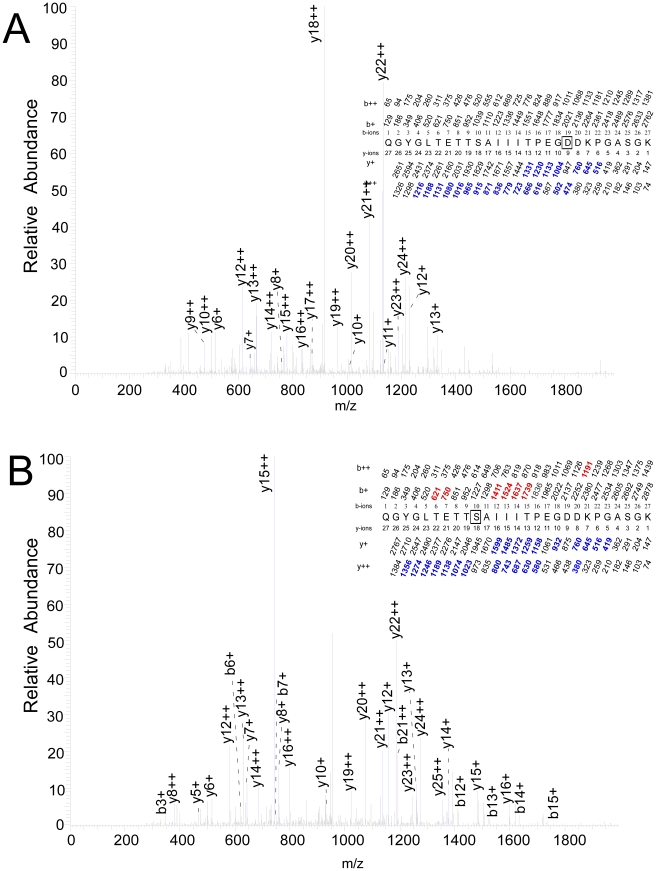
Identification of photolabeled residues in the peptide Gln-340 – Lys-366. Conditions are as in Fig. 4. **A.** 3-azibutanol (1 mM) photolabels Asp-358. **B.** TFD-benzyl alcohol (100 µM) photolabels Ser-349.

A second peptide that was photolabeled was a 27–residue tryptic peptide starting at Gln-340. Sequencing this peptide proved to be difficult. Neither digestion by chymotrypsin nor Asp-N produced a better situation. In some cases we could not unambiguously define the point of incorporation. When Japanese firefly luciferase was photolabeled with 1 mM 3-azibutanol after incubation for 200 ms with ATP, a triply charged tryptic peptide eluted from the HPLC at 20.3 min with a mass of 2779.9283 Da by FT–MS, corresponding to a photolabeled 27–residue peptide starting at Q340—QGYGLTETTSAIIITPEGDDKPGASGK. The MSMS spectrum of this peptide is shown in Fig. 5A. A sequence of doubly charged y-ions from y24++ to y9++, with the exception of y11++, was observed to be modified, suggesting that the fragment 358–DDKPGASGK is photolabeled. Consistent with this, a run of weaker singly charged y-ions was observed from y13 to y6, with the exception of y9. In this series all ions are modified except for y8, y7 and y6, narrowing the assignment of the photolabeled residue to Asp-358. The assignment based on the y+ ions is somewhat insecure because of their low amplitude. Thus, the evidence ruling out Glu-356 as a site of photolabeling rests on the large doubly charged y9++ and y10++ ions. The b ions had low intensity and were not used in this analysis. This assignment had an X_corr_ value of 5.8. Under the same conditions when luciferin was included no photolabeled peptides were observed.

Tryptic digestion of Japanese luciferase photolabeled with 100 µM TFD-benzyl alcohol after incubation for 200 ms with ATP yielded a triply charged photolabeled peptide of mass 2894.3869 that eluted at 24.8 min. It was identified as the same peptide as above, starting at Gln-340, photolabeled by a single TFD-benzyl alcohol. A complete series of doubly charged y-ions from y25++ to y19++ were all modified (Fig. 5B). Another series of y++ ions from y16++ to y12++ were unmodified. Thus, photoincorporation must be in either Ser-349 or Ala-350. This assignment is consistent with the weaker run of unmodified y+ ions from y16 to y12 and from y8 to y5. Based on a large body of photolabeling data with aromatic diazirines, in particular 3-(trifluoromethyl)-3-(m-iodophenyl) diazirine (TID), alanine has a very low probability of being photolabeled, so we conclude that Ser-349 is the residue photolabeled by TFD-benzyl alcohol. When luciferin and ATP were added simultaneously for 200 ms there was no photoincorporation into this peptide.

### Residues photolabeled on peptide Cys-284 – Lys-299

**Figure 6:**
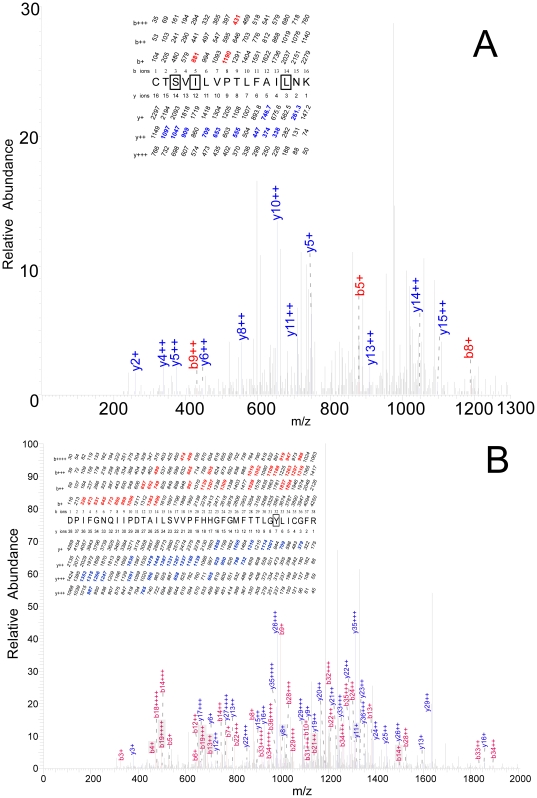
Identification of photolabeled residues in two peptides. Conditions are as in Fig. 4. **A.** TFD–benzyl alcohol (100 µM) photolabels Ser-314 in the peptide Cys-284 – Lys-299. **B.** 3-azibutanol (1 mM) photolabels Tyr-257 in the peptide Asp-226 – Arg-263.

In the time resolved experiments with 100 µM TFD–benzyl alcohol, the 16–residue peptide CTSVILVPTLFAILNK eluted at ∼25 minutes as a doubly charged ion with molecular weight of 2296.1277 Da by FT–MS. Unfortunately, it proved very difficult to sequence this peptide reliably. The best sequence (X_corr_ = 1.75) was observed for a triply labeled peptide in a run with ATP exposure for 200 ms. In a series of doubly charged y-ions, from y15++ to y10++, with the exception of y12++, one photolabel was lost between y14++ and y13++ and a second between y13++ and y11++. The final photolabel was still attached at y6++, y5++ and y4++, but likely not at y2+ (Fig. 6A). This data is consistent with photoincorporation at Ser-286, Ile-288 as well as at one of the C-terminal residues, 297–LNK. Of these three residues, photoselectivity favors Leu-297 and y2+ probably rules out Lys-299. This assignment of labeling was typical of many other lower quality spectra. Photoincorporation into this peptide was much more efficient (∼5×) than into the peptide in the previous section. It was often observed with 2 or 3 TFD–benzyl alcohol molecules photoincorporated.

### Residues photolabeled on peptide Asp-226 – Arg-263

This peptide was rarely seen when the Japanese luciferase was sequenced, but was observed in the American luciferase, possibly because 6 residues are not conserved. When American firefly luciferase was photolabeled with 1 mM 3-azibutanol after incubation for 200 ms with ATP, a quadruple charged tryptic peptide eluted from the HPLC at 32.5 min with a mass of 4266.1344 Da by FT–MS, corresponding to a photolabeled 38–residue peptide DPIFGNQIIPDTAILSVVPFHHGFGMFTTLGYLICGFR starting at Asp-226. This long peptide with 5 glycine and 3 proline residues was difficult to sequence entirely. A run of y-ions that all include a photolabel from y26++ to y12++ with the exception of y18++, y17++ and y14++, suggest that the photolabel is on one of the 12 residues at the N-terminal end of the peptide (Fig. 6B). A fairly complete run of modified b-ions from b28+++ to b36+++ except b30 & 33+++ with loss of label between b32+++ and b34+++, together with unmodified b33++++ and b34++++ ions is consistent with photoincorporation on Tyr-257. Although some of these peaks are small, this conclusion is reinforced by the fact that Tyr-257 is the only residue in the N-terminal dozen residues that aliphatic diazirines have been observed to react with [24].

### Minor labeling sites

A number of other peptides were photolabeled in an ATP–independent manner by azialkanols. These residues were invariably on the surface. For example, in a time–resolved photolabeling experiment with 1 mM 3-azibutanol in the presence of ATP and luciferin, luciferin–independent photolabeling was seen in the lid domain, primarily in Glu-499 and Tyr-304, and in a surface residue, Tyr-58. None of these residues are near the known binding sites for ATP or luciferin, and they were not photolabeled at 10 µM 3-azioctanol or 100 µM 3-azibutanol, so they represent low affinity nonspecific photoincorporation. Another surface residue, Tyr-18, was photolabeled by 1 mM 3-azibutanol in the absence but not presence of ATP. It was also observed at 10 µM 3-azioctanol and 100 µM 3-azibutanol, concentrations at which the other surface residues were not seen.

### Anesthetic potency

Loss of righting reflex was determined over the concentration range 5–100 µM TFD-benzyl alcohol. All animals recovered from anesthesia. The EC50 for loss of righting reflexes was 28±6.7 µM and the slope was 2.1±0.76.

## Discussion

### Conformation–dependent photoincorporation

3-Azioctanol inhibited the initial light emitting reaction of luciferase following the addition of luciferin and ATP, resembling other alkanols in this respect [16], [25]. It had an IC_50_ of 200 µM and a Hill coefficient of one (Fig. 2A & B). At equilibrium, the concentration dependence of overall photoincorporation of [^3^H]3-azioctanol into luciferase increased linearly with concentration in the presence of ATP, suggesting low affinity nonspecific binding, but in the absence of ATP it increased in a saturable manner suggesting interaction with one or more binding sites (Fig. 3). The latter interaction was of comparatively low apparent affinity (∼1 mM) compared to the IC_50_ for inhibition of the initial light emitting reaction (Fig. 2B). However, total photoincorporation of [^3^H]3-azioctanol more than doubled when ATP was added for only 200 ms. These observations suggest that when ATP interacts with apo-luciferase in the first few 100–milliseconds it converts the low affinity alcohol site into a site of higher affinity, but that it subsequently drives a conformation change that dramatically reduces the affinity of the site for alcohols (affinity increases in the order: ATP at equilibrium<no ATP<ATP at 200 ms).

Sequencing revealed a number of surface residues that were photolabeled in an ATP-independent manner (Results), as well as buried residues in which photolabeling was state dependent. The most robust ATP–dependent photoincorporation of azialkanols occurred at Glu-313 (3-azibutanol, 3- and 7-azioctanol) (Fig. 4A, 5B and S1). The modified peptide could be detected when luciferase was photolabeled at equilibrium in the absence of ATP at concentrations as low as 10 µM 3-azioctanol (Fig. S3) and 100 µM 3-azibutanol, concentrations at which all but one of the surface residues were not photolabeled. Although the sampling logic of the mass spectrometer prevents quantitative interpretation, the conditions of these experiments were all very similar so a qualitative comparison of the state–dependence of photoincorporation can be made by considering the frequency with which Glu-313 was observed to be photolabeled. The ranking of photoincorporation into Glu-313 in the three states is similar to that observed for [^3^H]3-azioctanol into luciferase itself (ATP at equilibrium<no ATP<ATP at 200 ms). Taken together with the observation that photoincorporation into Glu-313 tended to be reduced in the presence of luciferin, this evidence suggests that Glu-313 may be directly involved in alcohol inhibition of luciferase. Photoincorporation of TDF-benzyl alcohol into luciferase occurred at too low a frequency to reliably assess ATP–dependence.

Most of the surface residues showed no conformation dependence. However, there were two exceptions. First, azialkanols photolabeled Asp-358 in an ATP-dependent manner. The pattern of photolabeling was unusual. It was only observed in the absence of ATP, and unlike Glu-313, exposing to ATP for 200 ms abolished photolabeling by the azioctanols. The only exception was azibutanol that did photolabel after 200 ms of ATP exposure, and luciferin then abolished photolabeling. Asp-358 is a surface residue beyond the extension of the luciferin pocket that contains Glu-313 (its α-carbon is 11 Å from the ε-oxygen of Glu-313 Å). When the +DSLA (5′-O-[*N*-(dehydroluciferyl)- sulfamoyl]adenosine) and +ATP structures are superimposed, the position of the alpha carbons of Asp-358 are seen to differ by nearly 1 Å. Moreover, this region of the protein exhibits high b-factors (temperature factors) in all reported structures, suggesting considerable inherent mobility. Thus, this surface pocket appears to be sensitive to conformation but is unlikely to be involved in inhibition by alcohols. The second exceptional residue, Tyr-18, lies on the N-terminal strand that traverses the surface of luciferase. In all the published structures, the Cα of Tyr-18 has the same position, but in the bromoform bound American luciferase structure (1BA3.pdb) its side chain adopts a different rotamer. The crystal structures provide no explanation for the behavior of this residue. It remains possible that this part of luciferase adopts a different conformation in solution.

### Photolabeled sites in the luciferin–binding pocket

The location of photolabeled residues can be discussed in the context of several crystal structures [10], [11], [12], [17]. Of these, that of Japanese luciferase determined by Nakatsu et al [11] is the most complete, has the highest resolution, and was determined with various ligands bound [11], although they were unable to obtain crystals of the apo state. Of course, there is no crystal structure of the short-lived conformation we captured by photolabeling at 200 ms after adding ATP, but it is claimed that the concatenated ligand DSLA (Fig. 1B) that occupies both the luciferin and ATP binding sites emulates the active state. The structure with DSLA bound has by far the highest resolution (1.3 Å) of all published structures, and we will discuss the location of the photolabeled residues with respect to it (2D1S.pdb). The position of oxyluciferin in the binding pocket (2D1R.pdb [11]) is isosteric with the benzothiazole ring of DSLA (Fig. 1B). DSLA, regarded as a high-energy intermediate analogue, induces a ‘closed form’ of the active site, in which its benzothiazole ring is tightly sandwiched in a hydrophobic pocket, whereas the states before and after the reaction are in an open form [11].

**Figure 7:**
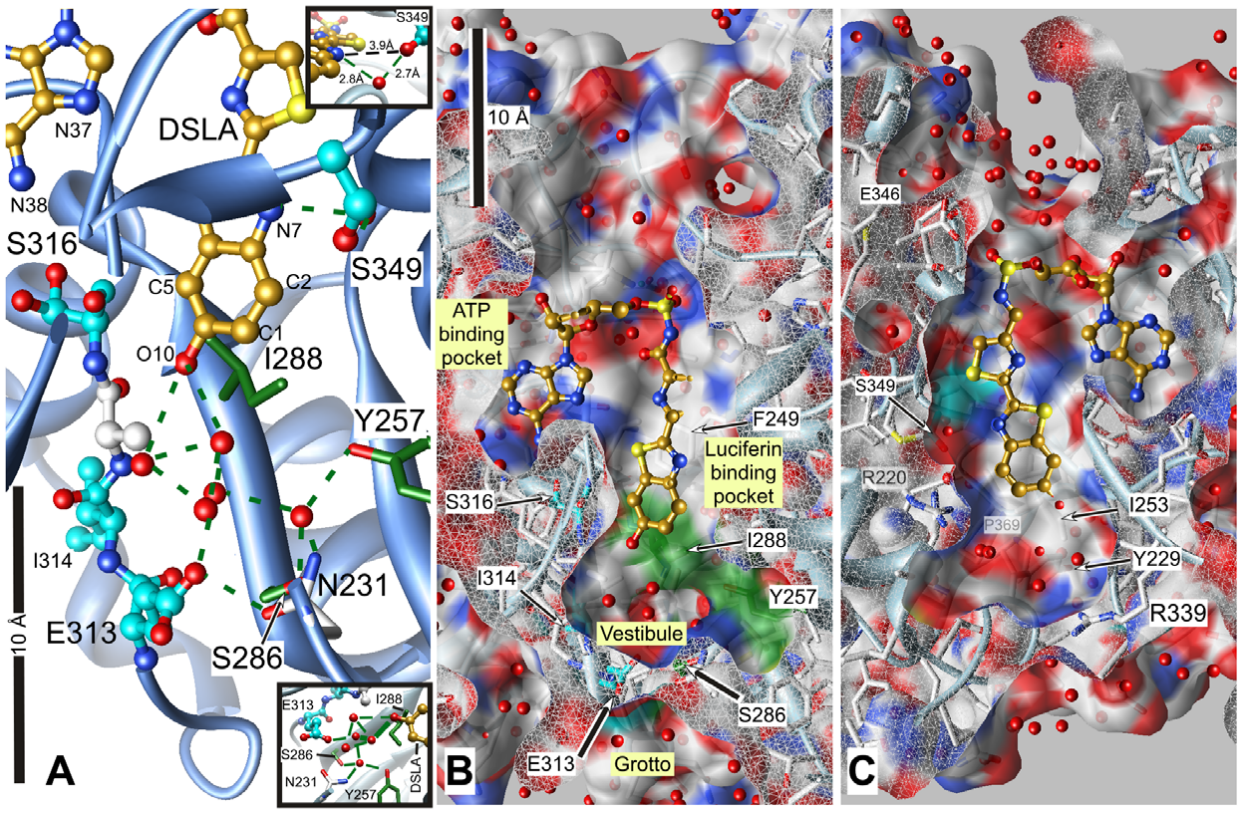
The structure of Japanese luciferase in the vicinity of the main photolabeled residues. The structure is shown as a cross section through the luciferin pocket with DSLA bound in the luciferin and ATP sites (2D1S.pdb). Panel A shows a close up view. Panel B is the same view zoomed out (note the 10 Å scale bars) to show the luciferin and ATP pockets. Panel C was obtained by rotating panel B 180° on the y-axis without scaling, viewing the pocket from the opposite side. Cross sections through the protein are capped in white mesh, revealing the residues behind. DSLA is shown with golden carbons. The main photolabeled residues are shown in ball & stick representation with cyan carbons; note that Ser-316 shows two rotamers with each oxygen assigned half occupancy. Important residues for the activity of luciferase that may have been photolabeled are shown with green carbons in stick representation. Hydrogen bonds to ligands and water are shown in panel A by green dashed lines. Surface colors are: white, carbon (except for photolabeled residues, cyan or green); blue, nitrogen; red, oxygen. The upper inset in panel A shows the interaction between Ser-349 and DSLA and the hydrogen bonded water. The lower inset shows the hydrogen bonding network linking the major residues interacting with DSLA through water molecules. Hydrogen bonds shown range from 2.5 to 3.0 Å in length.

At the competitive inhibition site within the luciferin–binding pocket, the photolabeled Ser-349 and Ser-316 are 10 Å apart and contact the benzothiazole ring of DSLA on opposite sides (Fig. 7A). The hydroxyl of Ser-349 is ∼4 Å from the benzothiazole ring (N7 & C2) and hydrogen bonds to N7 through an intermediate water (Fig. 7A, upper inset). The backbone carbonyl of Ser-316 is 3.8 Å from the C5 of the benzothiazole ring and 3.9 Å from its terminal oxygen (O10). C-terminal from Ser-316 (316–SGGAAPL–321), the backbone makes a hairpin turn at the second of the two glycines (Gly-318), which is in contact with both the adenosine ring of ATP and the benzothiazole ring of DLSA (not shown). Indeed, the separation of O10 of the benzothiazole ring from N37 of the adenosine ring is only 7.6 Å. In the +ATP structure, this strand has poor electron density and high b-factors, but once both binding pockets are occupied the electron density improves dramatically and the b-factors fall. Thus, ligand binding induces important changes in this turn, coupling the two binding pockets. Our emphasis on the dynamics in this region is supported by simulations using Gaussian and anisotropic network models that predict that anesthetics cause an increase in flexibility in this region [26].

Allosteric action can occur because the luciferin–binding pocket is not completely filled by luciferin or the benzothiazole ring of DSLA (Fig. 7B & C). The total length of the pocket is some 20 Å, and an unoccupied vestibule, 6–7 Å long extends beyond DSLA's terminal oxygen (O10). The vestibule is wider and has a more irregular shape than the luciferin–binding region. Ser-286 and Ile-288, which are likely photolabeled by TFD-benzyl alcohol, lie close to Glu-313. Together with the photolabeled Tyr-257, they line much of one side of the vestibule. As has been pointed out [11], the exact spatial relationship between these three residues varies with the conformation of luciferase. Upon activation, the side chain of Ile-288 rotates and moves towards luciferin, helping to create a tightly packed hydrophobic environment for light emission, and Ser-286 releases its hydrogen bond with Glu-313, and instead hydrogen bonds to Tyr-257 via a water molecule (Fig. 7A, lower inset). In consequence, a network of hydrogen bonds involving five water molecules couples Asn-231, Tyr-257, Glu-313, Ser-286 and the O10 oxygen of DSLA. Recent theoretical calculations of the activated state of luciferin have placed an emphasis on the state of the O10 of luciferin and its associated hydrogen–bonded network of water molecules whose configuration depends on the orientation of the surrounding residues including Glu-313 [27], [28]. The allosteric action of anesthetics on luciferase, which is revealed at high luciferin concentration [17], may well involve displacement of these waters as well as rearrangement of surrounding residues.

**Figure 8:**
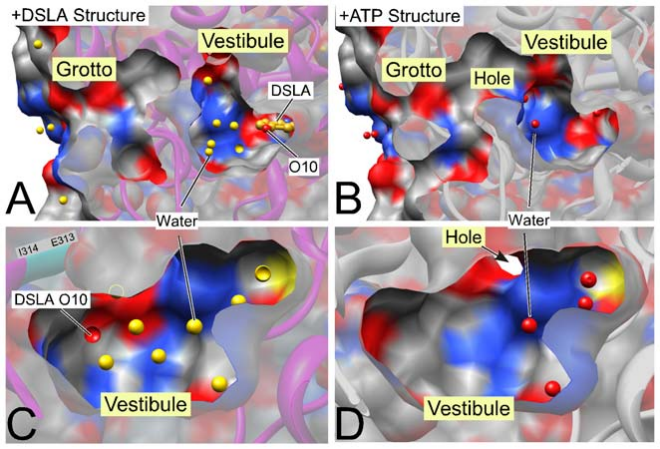
Ligand-induced changes in the vestibule and grotto region of luciferase. The “active” DSLA bound (left, 2D1S.pdb) and ATP bound (right, 2D1Q.pdb) structures are compared from the same viewing points. Upper panels show a cross section through the vestibule and grotto region beyond the luciferin binding pocket (compare Fig. 7). The lower panels show the vestibule as viewed from the luciferin pocket; the left panel shows the O10 of DLSA for orientation. Water molecules are shown in red in the +ATP structure and in gold in the DSLA structure (one of the eight water molecules is partially hidden in panel C and is indicated with a circle). The cross-section surface capping is semitransparent. Surface coloring is grey for carbon, red for oxygen and blue for nitrogen. DSLA (gold carbons in A) is a surrogate for luciferin (Fig. 1B). Similar changes are seen in American luciferase (3IES.pdb and 3IEP.pdb).

In addition, Glu-313 is in the interface between the vestibule and a complex surface pocket, which we will call the grotto (Fig. 7B). The vestibule and grotto are separated by the side chains of a number of residues in the activated, DSLA–bound state, but in the equilibrium +ATP state, the above rearrangements open up the vestibule to the grotto (Fig. 8), perhaps contributing to the lower affinity for anesthetics in this state.

### Nature of the anesthetic binding pocket

The region close to the benzothiazole ring of DSLA in the luciferin pocket is largely apolar on one side, with Ile-288 and Phe-249 underlying the ring (Fig. 7B). The other half of the surface is more polar, being exposed to the backbone of the strand 341–GYGL–344 and to the side chain of Ile-253 (Fig. 7C). In the vestibule, polar residues predominate. This mix of polarity is expected for general anesthetic binding pockets in proteins [29].

The vestibule in the DSLA structure is filled by eight water molecules, whereas in the +ATP structure (2D1Q.pdb) it contains only 4 water molecules (Fig. 8). These waters are category two waters in the Ringe classification, meaning that they are observed in some but not all conformations of the protein [30], and their displacement by an anesthetic would more than offset the entropic cost of confining the anesthetic to the binding pocket. Because there are more waters in the DSLA “active” structure than in the +ATP structure, the entropic contribution from their displacement would favor binding to the former state, as is observed (Fig. 2). A similar ratio of waters is found when the active and Apo structures of the American luciferase are compared (3IES.pdb vs. 3IEP.pdb). Thus, the conformation with the most hydration in the vestibule is also the one with highest affinity for alcohols.

A survey of structures with anesthetics bound, concludes in addition, that some waters may remain in the pocket and compensate for imperfectly fitting anesthetics [29]. Such a mechanism might allow the pocket and vestibule to accommodate ligands of variable structure, which would be consistent with the wide range of agents that inhibit luciferase [7], [9], [25], [31]. Indeed, even the oxyluciferin– and DSLA–occupied pockets contain a water molecule hydrogen–bonded to Ser-349 and the benzothiazole ring's nitrogen.

A feature of many of the photolabeled residues (shown as bold) is their proximity to small residues, such as glycine and alanine, that permit some local flexibility (312 –V**EI**A**S**GGA–; 347 –T**S**AI–; 357–G**D**DK–; 256–G**Y**LI–; for clarity alanine and glycine are marked in blue). Crystallography provides the mean equilibrium location of atoms usually at liquid nitrogen temperature, but at physiological temperatures and in solution considerably more motion must occur. A measure of relative mobility within the crystal is given by the b-factors, which tend to increase as one moves from residues proximal to DSLA to those in the vestibule and beyond that into the grotto region. For example, the side chain b-factor of Ser-349 next to DSLA is 4 Å^2^ whereas those of Glu-313 and of Asp-358 in the same structure are respectively 13 Å^2^ and 19 Å^2^. Furthermore, mobility can vary with the state induced by ligand binding. For example, in the DSLA bound state, the electron density of the residues following Glu-313 is good, but with only one (the ATP–bound structure) or no (the apo structure) ligand bound it is poor (high b-factor) [11] [12]. It is possible that the mobility in this region contributes to the diversity of general anesthetic structures that may inhibit luciferase.

### Relationship of photolabeled residues to other ligands bound at equilibrium

**Figure 9:**
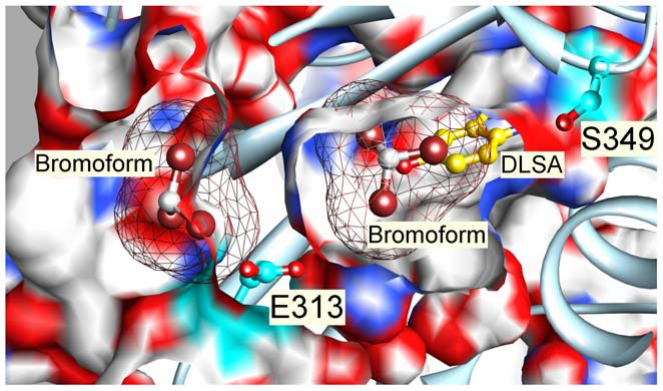
Relationship of photolabeled residues to other ligands bound. The structure of American luciferase with two bromoform molecules bound (1BA3.pdb; bromine atoms are brown) was superimposed on the DSLA–bound Japanese luciferase structure (2D1S.pdb). A cross section of 2D1S is shown without surface capping in a surfaced ribbon diagram (light blue) with the photolabeled residues shown with cyan carbons. DLSA is shown with gold carbons. The surface of the bromoforms is shown in mesh representation. The carbon atom of the inner bromoform (on the right), is centered within 2 Å of the terminal oxygen (O10) of DSLA and overlaps C1, C5 and C6 of the benzothiazole ring of DSLA (see Fig. 7 for numbering). The outer bromoform (left) is situated in the grotto region 2.7 Å from Glu-313. It clashes sterically with the superimposed Japanese luciferase structure.

There is an Apo structure of the American luciferase (1BA3.pdb 2.2 Å resolution [17]) with two molecules of the general anesthetic bromoform bound with half occupancy at each site. Their carbons are separated by 9.3 Å. When the high resolution DSLA–bound (2D1S.pdb) and the bromoform–bound structures are superimposed (Fig. 9), the carbon atom of the inner bromoform, is centered within 2 Å of the terminal oxygen (O10) of DSLA, and the bromines overlap C1, C5 and C6 of the benzothiazole ring of DSLA. Glu-313 is between the two bromoforms, ∼5 Å from each carbon. The inner bromoform is positioned close to residues that we photolabeled, but the outer bromoform occupies a complex surface pocket, the grotto, separated from the luciferin pocket's vestibule by the side chain of Glu-313. The inner bromoform is well placed to compete with luciferin, and the outer may account for the allosteric inhibition observed at saturating luciferin concentrations that overcome competitive inhibition [17]. If the DSLA structure really represents the activated state, then in addition to competition with luciferin, modest steric clashes must be overcome in the binding sites for bromoform (Fig. 9).

In the more recent structures of a truncated American luciferase, two Apo forms were reported. One (3IER.pdb, 2.05 Å) had polyethylene glycol modeled in the luciferin pocket. The second Apo structure (3IEP.pdb. 2.1 Å) lacked this molecule. However, we observed excess electron density in the vestibule region of 3IEP.pdb with a shape similar to ethylene glycol. The insets in Fig. S4 show the result of placing such a molecule by eye. This molecule, like bromoform, overlaps the terminal region of PTC-124-AMP, a ligand analogous to DSLA in the Japanese structure. (Fig. S4).

### Conclusions

Our findings are consistent with inhibition of luciferase occurring at an alcohol–binding locus that has the highest affinity in the activated state. In this state, time–resolved photolabeling places the alcohols both in the region of the luciferin pocket that is intimately associated with luciferin (competitive site) and in the vestibule in the unoccupied extension of that pocket (allosteric site). In addition, it is possible that occupancy also occurs in a surface pocket or grotto adjacent to the vestibule. This is broadly consistent with the notion that inhibition has both a competitive and a noncompetitive component [17]. That the sites photolabeled at 200 ms after adding ATP were the same ones that were photolabeled with no ATP present supports an allosteric model in which the affinity but not the location of the sites varies with the conformation of luciferase.

## Supporting Information

Figure S1 **Identification of residues photolabeled by 7-azioctanol (100 µM) in the peptide Tyr-306– Lys-324.** After photolabeling at equilibrium in the absence of ATP, Japanese firefly luciferase was digested with trypsin and subject to HPLC-MSMS as described in the Methods section. An 18–residue tryptic photolabeled peptide bearing a single 128 Da modification was identified as YDLSNLVEIASGGAPLSK starting at Y306. The site of photoincorporation for 7-azioctanol was inferred from this MSMS spectrum. In the inset, the predicted charge/mass ratios of ions with an intact N-terminus (b-ions) or C-terminus (y-ions) are shown above and below the sequence, respectively, with the indicated charge. The photolabeled residue is boxed and the experimentally observed values are colored (b-ions in red and y-ions in blue) and in bold and their position indicated on the spectrum. A run of strong y-ions from y14+ to y4+ with loss of label between y11+ and y10+ identify Glu-313 as the photolabeled residue. This is confirmed by the b+ ions.
(TIF)

Figure S2 **Identification of residues photolabeled by 3-azibutanol (1 mM) in the peptide Tyr-306– Lys-324.** After treatment as in Fig. S1, an 18–residue tryptic photolabeled peptide bearing a single 72 Da modification was identified as YDLSNLVEIASGGAPLSK starting at Y306. Loss of label was observed between y11+ and y10+ in a run of strong y-ions, confirming Glu-313 as the photolabeled residue. This conclusion is also consistent with the b-ions.
(TIF)

Figure S3 **Identification of residues photolabeled by 3-azioctanol (10 µM) in the peptide Ser-300–Lys324 from American firefly luciferase.** After treatment as in Fig. S1, a 24–residue tryptic photolabeled peptide bearing a single 128 Da modification was identified as STLIDKYDLSNLHEIASGGAPLSK starting at S300. To avoid crowding, some ions are not labeled in the spectrum. All the observed y-ions were modified, showing that photolabeling is on or N-terminal to Glu-313 (Japanese numbering). This conclusion is confirmed by a run of b-ions from b14+ to b3+, with the exception of b10+. The strong unmodified b++-ions, b16 & 17++, b20++ and b22 & 23++ place the label on Glu-313 or Ile-314. Aliphatic diazirines do not react with isoleucine, so we conclude that Glu-313 is photolabeled, as was found at 100 µM 3-azioctanol (Fig. S1).
(TIF)

Figure S4 **Ethylene glycol and bromoform occupy similar positions in the vestibule.** The structure of American luciferase with PTC-124-AMP bound (3IES.pdb) with the two bromoform molecules from 1BA3.pdb superimposed, and the ethylene glycol from 3IEP.pdb (see below). A cross section of 3IES is shown with surface capping in a surfaced ribbon diagram (magenta) with the photolabeled residues shown with cyan carbons. PTC-124-AMP is shown with gold carbons. We observed excess density at the 3σ level in the vestibule region of the Apo structure (3IEP.pdb). We modeled ethylene glycol by eye in this density using Coot [23]. The insets show the electron density for this region. Left inset shows the environment of the ethylene glycol with the 2F_o_ electron density at 1σ shown in green. The right inset shows the ethylene glycol in detail with the difference map (F_o_−F_c_) in magenta at 3σ.
(TIF)
