# Supplementary figures and images for: The Location and Nature of General Anesthetic Binding Sites on the Active Conformation of Firefly Luciferase; A Time Resolved Photolabeling Study
Source: PLoS One. 2012 Jan 17;7(1):e29854. doi: 10.1371/journal.pone.0029854 (PMC3260189; doi:10.1371/journal.pone.0029854)

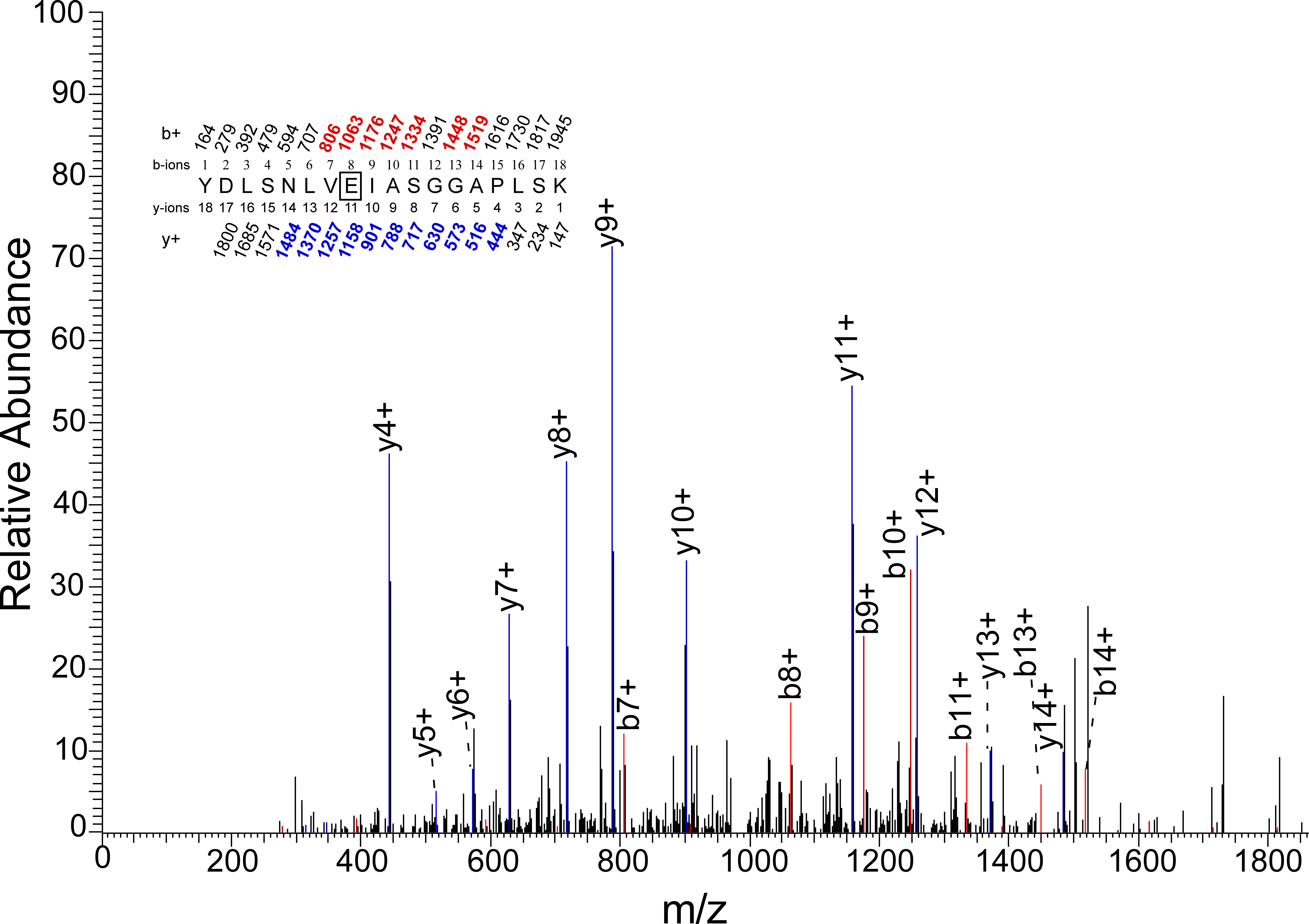

Supplement: Figure S1 — Identification of residues photolabeled by 7-azioctanol (100 µM) in the peptide Tyr-306– Lys-324. After photolabeling at equilibrium in the absence of ATP, Japanese firefly luciferase was digested with trypsin and subject to HPLC-MSMS as described in the Methods section. An 18–residue tryptic photolabeled peptide bearing a single 128 Da modification was identified as YDLSNLVEIASGGAPLSK starting at Y306. The site of photoincorporation for 7-azioctanol was inferred from this MSMS spectrum. In the inset, the predicted charge/mass ratios of ions with an intact N-terminus (b-ions) or C-terminus (y-ions) are shown above and below the sequence, respectively, with the indicated charge. The photolabeled residue is boxed and the experimentally observed values are colored (b-ions in red and y-ions in blue) and in bold and their position indicated on the spectrum. A run of strong y-ions from y14+ to y4+ with loss of label between y11+ and y10+ identify Glu-313 as the photolabeled residue. This is confirmed by the b+ ions. (TIF) [file pone.0029854.s001.tif]

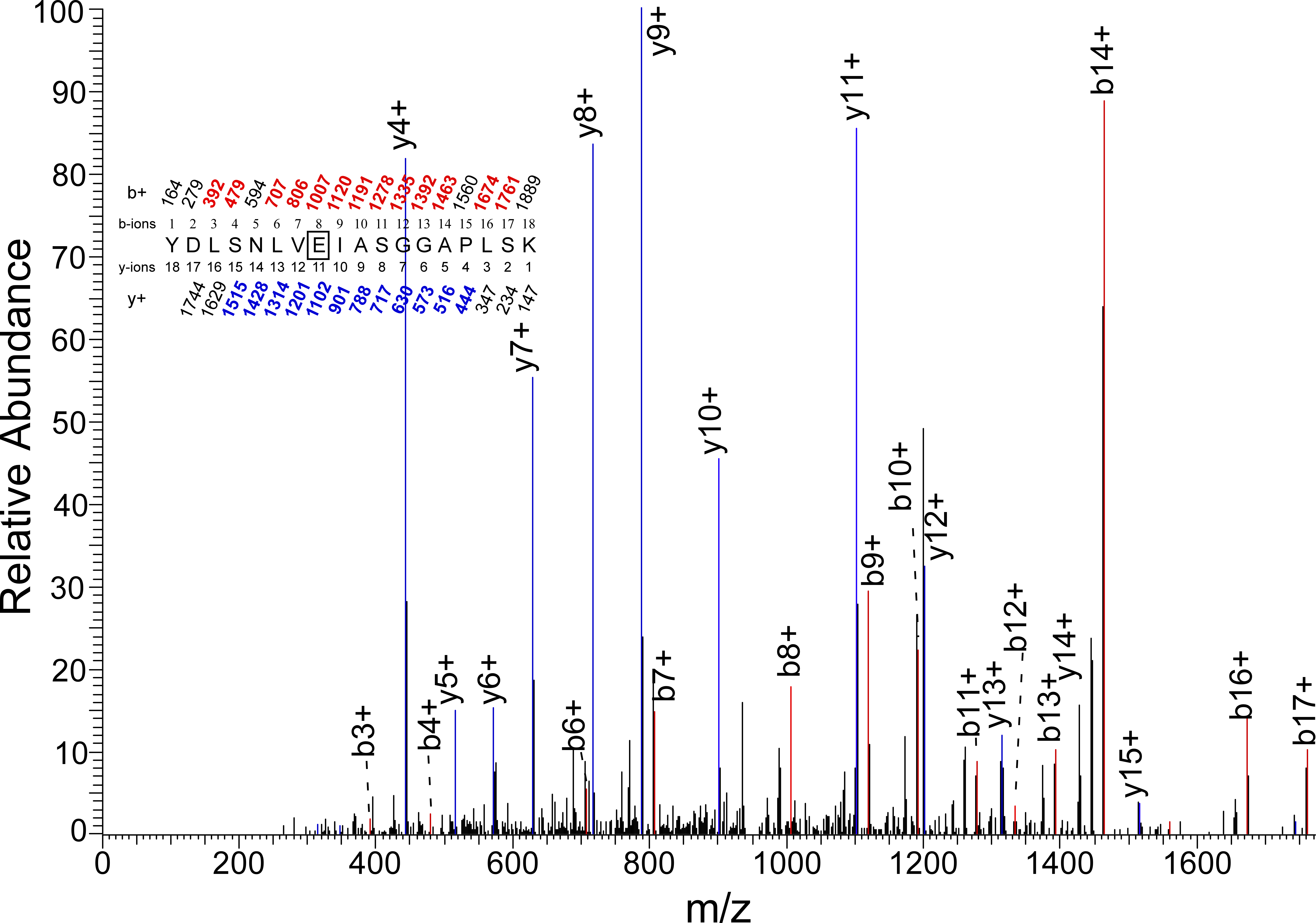

Supplement: Figure S2 — Identification of residues photolabeled by 3-azibutanol (1 mM) in the peptide Tyr-306– Lys-324. After treatment as in Fig. S1, an 18–residue tryptic photolabeled peptide bearing a single 72 Da modification was identified as YDLSNLVEIASGGAPLSK starting at Y306. Loss of label was observed between y11+ and y10+ in a run of strong y-ions, confirming Glu-313 as the photolabeled residue. This conclusion is also consistent with the b-ions. (TIF) [file pone.0029854.s002.tif]

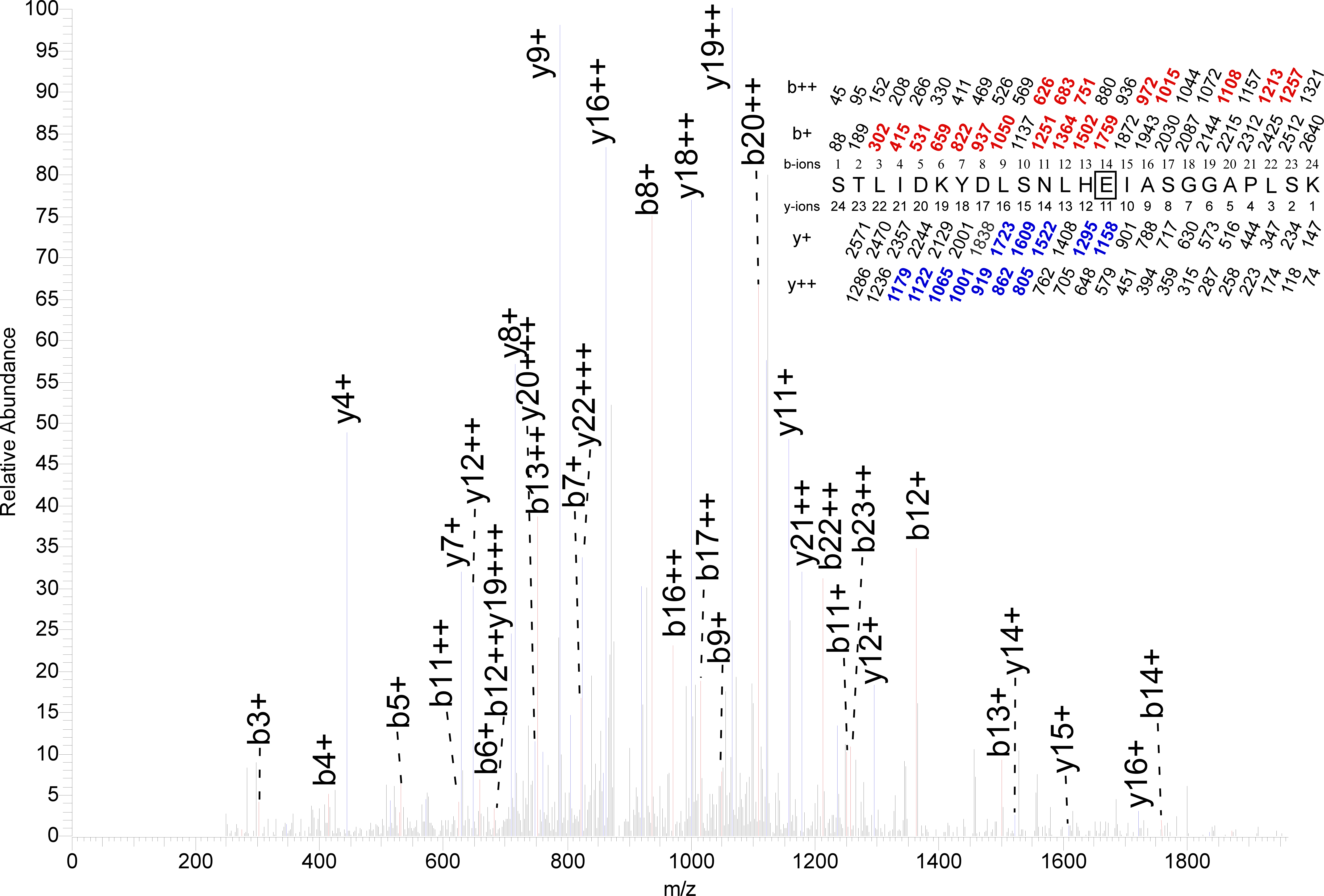

Supplement: Figure S3 — Identification of residues photolabeled by 3-azioctanol (10 µM) in the peptide Ser-300–Lys324 from American firefly luciferase. After treatment as in Fig. S1, a 24–residue tryptic photolabeled peptide bearing a single 128 Da modification was identified as STLIDKYDLSNLHEIASGGAPLSK starting at S300. To avoid crowding, some ions are not labeled in the spectrum. All the observed y-ions were modified, showing that photolabeling is on or N-terminal to Glu-313 (Japanese numbering). This conclusion is confirmed by a run of b-ions from b14+ to b3+, with the exception of b10+. The strong unmodified b++-ions, b16 & 17++, b20++ and b22 & 23++ place the label on Glu-313 or Ile-314. Aliphatic diazirines do not react with isoleucine, so we conclude that Glu-313 is photolabeled, as was found at 100 µM 3-azioctanol (Fig. S1). (TIF) [file pone.0029854.s003.tif]

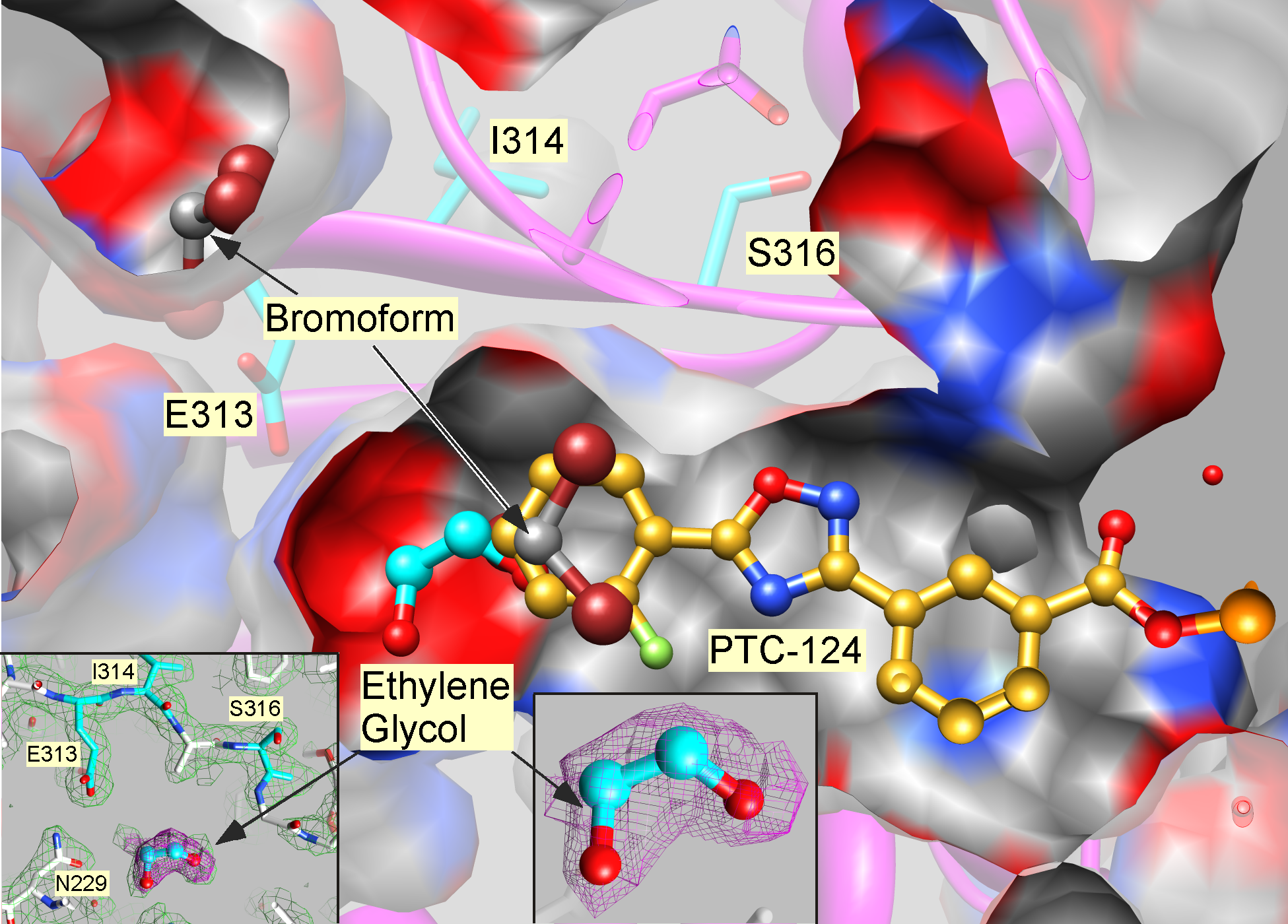

Supplement: Figure S4 — Ethylene glycol and bromoform occupy similar positions in the vestibule. The structure of American luciferase with PTC-124-AMP bound (3IES.pdb) with the two bromoform molecules from 1BA3.pdb superimposed, and the ethylene glycol from 3IEP.pdb (see below). A cross section of 3IES is shown with surface capping in a surfaced ribbon diagram (magenta) with the photolabeled residues shown with cyan carbons. PTC-124-AMP is shown with gold carbons. We observed excess density at the 3σ level in the vestibule region of the Apo structure (3IEP.pdb). We modeled ethylene glycol by eye in this density using Coot [23]. The insets show the electron density for this region. Left inset shows the environment of the ethylene glycol with the 2Fo electron density at 1σ shown in green. The right inset shows the ethylene glycol in detail with the difference map (Fo−Fc) in magenta at 3σ. (TIF) [file pone.0029854.s004.tif]
